# Insertion/Deletion Polymorphisms in the *ΔNp63* Promoter Are a Risk Factor for Bladder Exstrophy Epispadias Complex

**DOI:** 10.1371/journal.pgen.1003070

**Published:** 2012-12-20

**Authors:** Simon Wilkins, Ke Wei Zhang, Istiak Mahfuz, Renaud Quantin, Nancy D'Cruz, John Hutson, Michael Ee, Darius Bagli, Karen Aitken, Fion Nga-Yin Fong, Patrick Kwok-Shing Ng, Stephen Kwok-Wing Tsui, Wendy Yin-Wan Fung, Tahmina Banu, Atul Thakre, Kaid Johar, Enrique Jaureguizar, Long Li, Wei Cheng

**Affiliations:** 1 Monash Institute of Medical Research, Faculty of Medicine, Nursing, and Health Sciences, Monash University, Melbourne, Australia; 2 Department of Paediatric Urology, University of Melbourne, Melbourne, Australia; 3 Women's and Children's Clinical Services, Royal Hobart Hospital, Hobart, Australia; 4 Division of Urology, Hospital for Sick Children and University of Toronto, Toronto, Canada; 5 School of Biomedical Sciences, The Chinese University of Hong Kong, Hong Kong, China; 6 Department of Pediatric Surgery, Chittagong Medical College and Hospital, Chittagong, Bangladesh; 7 Iladevi Cataract and Intraocular Lens Research Centre, Civil Hospital, Ahmedabad, India; 8 Department of Urology, Hospital Universitario La Paz, Madrid, Spain; 9 Department of Surgery, Capital Institute of Pediatrics, Beijing, China; 10 Department of Paediatrics, Department of Surgery, Southern Medical School, Faculty of Medicine, Nursing, and Health Sciences, Monash University, Melbourne, Australia; 11 Department of Paediatric Surgery, Monash Children's, Southern Health, Melbourne, Australia; University of Pennsylvania, United States of America

## Abstract

Bladder exstrophy epispadias complex (BEEC) is a severe congenital anomaly; however, the genetic and molecular mechanisms underlying the formation of BEEC remain unclear. *TP63*, a member of *TP53* tumor suppressor gene family, is expressed in bladder urothelium and skin over the external genitalia during mammalian development. It plays a role in bladder development. We have previously shown that *p63^−/−^* mouse embryos developed a bladder exstrophy phenotype identical to human BEEC. We hypothesised that *TP63* is involved in human BEEC pathogenesis. RNA was extracted from BEEC foreskin specimens and, as in mice, *ΔNp63* was the predominant *p63* isoform. *ΔNp63* expression in the foreskin and bladder epithelium of BEEC patients was reduced. DNA was sequenced from 163 BEEC patients and 285 ethnicity-matched controls. No exon mutations were detected. Sequencing of the *ΔNp63* promoter showed 7 single nucleotide polymorphisms and 4 insertion/deletion (indel) polymorphisms. Indel polymorphisms were associated with an increased risk of BEEC. Significantly the sites of indel polymorphisms differed between Caucasian and non-Caucasian populations. A 12-base-pair deletion was associated with an increased risk with only Caucasian patients (p = 0.0052 Odds Ratio (OR) = 18.33), whereas a 4-base-pair insertion was only associated with non-Caucasian patients (p = 0.0259 OR = 4.583). We found a consistent and statistically significant reduction in transcriptional efficiencies of the promoter sequences containing indel polymorphisms in luciferase assays. These findings suggest that indel polymorphisms of the *ΔNp63* promoter lead to a reduction in p63 expression, which could lead to BEEC.

## Author Summary

Bladder exstrophy epispadias complex is a severe congenital abnormality. The affected babies' bladders are born open, leaking urine constantly. Treatment involves multiple major reconstructive surgeries and the need for lifelong care for the complications of the disease. Although a number of studies have suggested a genetic cause of the disease, the genetic and molecular mechanism underlying the formation of BEEC remains unknown. One gene, *TP63*, plays a crucial role in the early bladder development. Two different genetic promoters of *TP63* produce different forms of the protein with opposing properties. We have shown mice lacking *p63* displayed a deformity complex identical to human BEEC. There are no genetic mutations in the p63 protein in BEEC, so genetic variants in the promoter could alter protein expression. Our hypothesis was that loss of p63 expression due to sequence polymorphisms in a promoter is a risk factor for BEEC. We found promoter sequence variants that were statistically associated with the disease and the sequence variant location varied between Caucasian and non-Caucasian patients. This is particularly important as Caucasian populations have a higher risk of BEEC. These findings provide an explanation of BECC and a base for further study of *TP63* related genes in this disease.

## Introduction

Bladder exstrophy epispadias complex (BEEC; MIM600057) is a serious congenital anomaly present in 1 in 36,000 live births [1]. BEEC is manifested as a cluster of ventral midline defects including: 1) ventral bladder and abdominal wall defects, 2) epispadias (split external genitalia), 3) separation of the pubic bones and the rectus abdominis muscles, 4) exomphalos, and 5), ventrally displaced anus [2]. Treatment of BEEC requires a series of major reconstructive surgeries with high morbidity rate. Without treatment, the affected babies continuously leak urine through the bladder defects, resulting in skin excoriation, a progressive decline in renal function, a constant stench, and severe psychosocial strain for both patients and parents. Untreated BEEC patients develop chronic bladder mucosal irritation and have a 700-fold increased chance of developing bladder cancer. The genetic and molecular mechanisms underlying the formation of BEEC remain unclear. To date, studies have shown an increased incidence of BEEC in children of older mothers [3], in Caucasian populations [4], and a familial genetic component of pathogenesis [5]. Chromosomal abnormalities have been suggested as possible cause of BEEC and a genome wide linkage study suggested more than one gene was involved [6]. Among siblings and offspring of BEEC patients, the risk of BEEC increase dramatically from 1 in 10,000–50,000 to 1 in 100 and 1 in 70 respectively, representing a 500-fold increase in incidence [7]. The concordance rate among the monozygotic twins is much higher than that of dizygotic twins (62% vs. 11%), representing a 4500-fold increase in incidence compared to that of the general population [8]. Brought together these data clearly suggest a genetic component in BEEC pathogenesis.

A member of the p53 tumor suppressor family, the protein p63, is expressed in all stratified epithelia, including the bladder urothelium and the skin overlying the external genitalia during development [9]. The protein p63 plays a key role in initiating epithelial stratification during development [10]. Expression of *TP63* is regulated by two promoters, *TAp63* and *ΔNp63*, located upstream to exon 1 and exon 3 respectively [11]. *TAp63* protein isotypes are pro-apoptotic whereas *ΔNp63* isotypes are anti-apoptotic and both isotypes may compete for the same set of target genes [11]. Mouse embryos lacking *p63* have thin, non-stratified skin, a short tail, truncated limbs, and cleft palate and are perinatally fatal [11], [12]. *p63* is required for stratification of epithelia, including the bladder urothelium [10]; furthermore the epithelial-mesenchymal interaction is instrumental in bladder mesenchymal (smooth muscle) development [13]. We have demonstrated that *p63^−/−^* mouse embryos exhibit ventral midline defects identical to those of human BEEC, including ventral bladder and abdominal wall defects, separation of external genitalia, separation of pubic bones and rectus abdominis muscles, exomphalos, and ventrally translocated anus [9]. *p63* is expressed in the bladder urothelium during development, *TAp63* at an earlier stage (E9–11) and *ΔNp63* later (E11–19). *ΔNp63* is preferentially expressed along the ventral midline in the epithelium overlying the genital tubercle and ventral bladder. Moreover, the apoptotic activity of ventral urothelium of *p63^−/−^* bladder is markedly increased whereas cell proliferation is much reduced. We believe that bladder epithelial apoptosis and failure of induction of the adjacent mesenchyme lead to the development of a BEEC-like phenotype in mice [9]. This study investigates whether the loss of p63 expression due to genetic variants in the *ΔNp63* is a risk factor for BEEC. In this study we show the expression of the two different *TP63* promoters in BEEC patient tissue, and explore sequence of the *ΔNp63* promoter in BEEC patients and controls. Our studies suggest that *ΔNp63* is the dominant promoter in human tissue and its expression is significantly reduced in BEEC tissue in early bladder formation. We also find many sequence variants within the *ΔNp63* promoter and three insertion/deletion polymorphisms were significantly associated with increased risk of BEEC. Furthermore the sites of these polymorphisms varied between Caucasian and non-Caucasian patients.

## Results/Discussion

**Figure 1:**
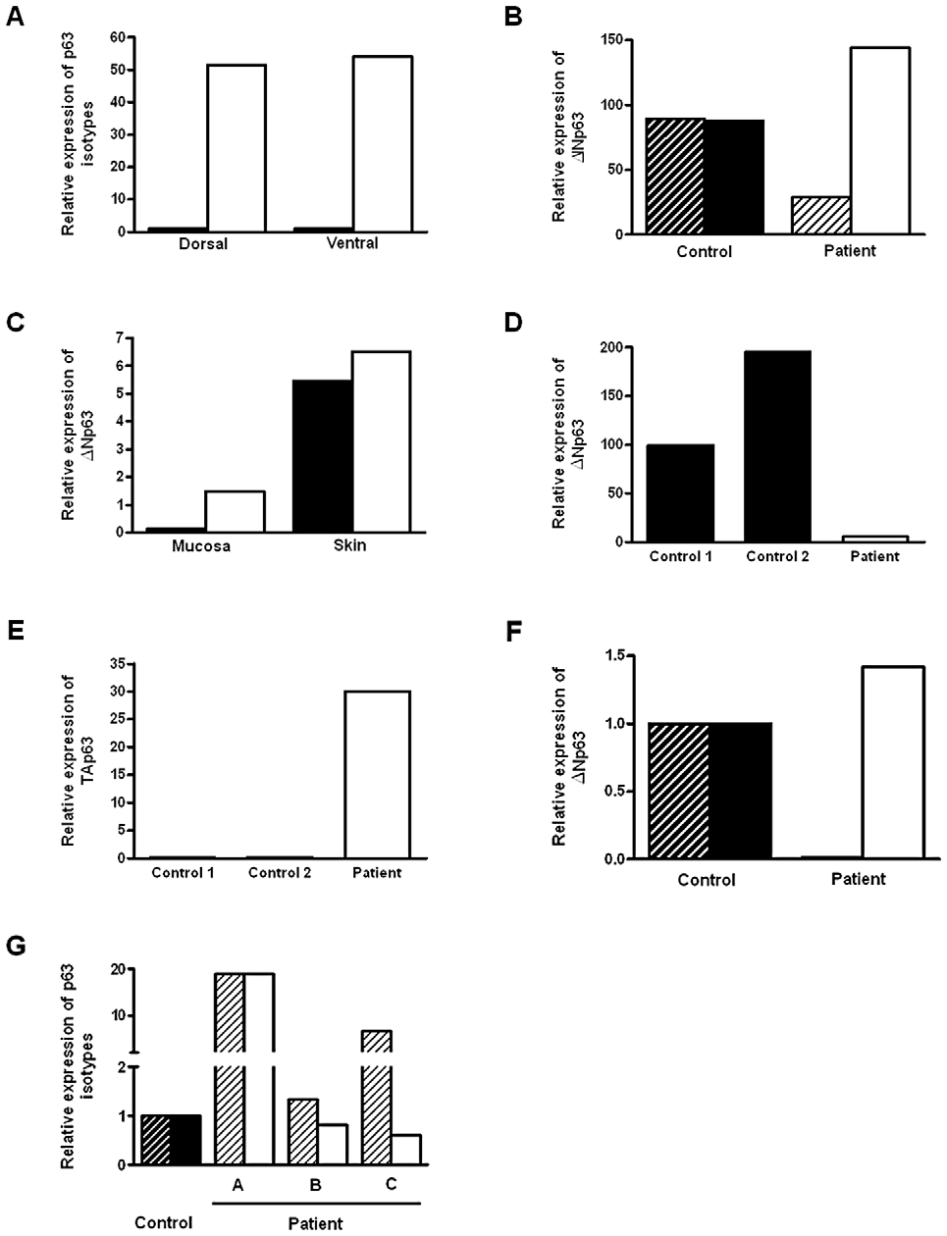
Expression of *p63* isoforms in BEEC patient tissue and controls. (A) Real-time qPCR of *ΔNp63* (white column) and *TAp63* (black column) expression dorsal and ventral foreskin from normal control (2 yr. male). (B) Real time qPCR of *ΔNp63* expression dorsal (black hatched column) and ventral foreskin (black column) from 2 normal controls and dorsal (hatched column) and ventral foreskin (white column) from a BEEC patient. (C) Real-time qPCR of *ΔNp63* expression in from BEEC patient (8 yr. male) dorsal (black column) and ventral (white column) foreskin mucosa and skin. (D) Real-time qPCR showing reduced *ΔNp63* mRNA expression in BEEC bladder epithelium compared with 2 normal controls. (E) Increased *TAp63* mRNA expression in BEEC bladder epithelium compared with 2 normal controls. (F) Real-time qPCR showing reduced *ΔNp63* mRNA and increased *TAp63* mRNA expression in bladder mucosa from a Caucasian BEEC patient (<1 yr. male) compared with normal controls (normalized to 1). (G) Real-time qPCR showing *ΔNp63* and *TAp63* mRNA expression in bladder mucosa from a three Caucasian BEEC patients (A, 13 yr. male; B, 14 yr. female; C, 6 yr. male) compared with normal controls (normalized to 1).

To establish if *TP63* plays a role in human bladder exstrophy, we first confirmed by real time-PCR that normal human foreskin (from circumcision) predominantly expresses *ΔNp63* mRNA, whereas the *TAp63* isoform was expressed at lower levels (Figure 1A). Compared with normal controls, the *ΔNp63* expression in the dorsal foreskin (adjacent to the epispadias) was decreased and also decreased compared to the patient's own ventral foreskin (opposite side) (Figure 1B). Down-regulation of *ΔNp63* appears to be mainly in the foreskin mucosa (non-keratinized epithelium) in an 8-year old BEEC patient (Figure 1C). Strikingly, in a tissue sample taken from the ventral bladder of a 2 day old Caucasian BEEC patient, *ΔNp63* expression was decreased whereas *TAp63* was increased compared with normal controls (Figure 1D and 1E). Tissue taken from the bladder of a<1 year old Caucasian BEEC patient also had deceased *ΔNp63* and increased *TAp63* compared with normal controls (Figure 1F). Expression of *ΔNp63* and *TAp63* in older Caucasian BEEC patients showed more varied expression (Figure 1G). The older patients haven undergone Mitroffanof procedures (using appendix as a conduit for bladder catheterization) and bladder augmentation (using small bowel patch to increase bladder volume). The irritation from small bowel mucus secretion and various degree of cystitis (from bacteria introduced by catheters) may affect *TP63* expression in the urothelia sampled. The genotypes of patient samples from Figure 1D–1G are shown in Table 1. We found that *ΔNp63* expression in some of the BEEC patients' urothelia is reduced. Although the post-natal expression does not necessarily represent that of the developing embryos and the sample size of post-natal bladder urothelia will not be large enough to draw statistically convincing conclusion, our data does demonstrate that *TP63* is expressed in neonatal bladder urothelium and neonatal foreskin of normal individuals and BEEC patients. The possible reduced *ΔNp63* expression during development could be one of the possible mechanisms of BEEC pathogenesis.

**Table 1:** Genotypes of BEEC patient samples used in real-time expression experiments.

	Genotype of patient samples used in real-time expression				
Promoter Position	Figure 1D/1E	Figure 1F	Figure 1GPatient A	Figure 1GPatient B	Figure 1GPatient C
−2657	A/A	A/A	A/A	A/A	A/A
−2651	C/C	C/C	C/C	C/T	C/T
−2293 to −2282	TCCAGAATCTT/-	TCCAGAATCTT/TCCAGAATCTT	TCCAGAATCTT/TCCAGAATCTT	TCCAGAATCTT/-	TCCAGAATCTT/-
−1944	C/C	C/C	C/C	C/C	C/C
−1287	T/-	T/T	-/-	T/-	T/-
−1209	T/T	T/T	C/C	C/C	C/C
−1059	C/C	C/C	C/C	C/C	C/C
−71	AGAG/-	AGAG/AGAG	AGAG/-	AGAG/AGAG	AGAG/-

Our data corroborate a recently published study where three out of the five BEEC patients had no *ΔNp63* expression detected in their bladders [14]. Our results showing reduced levels of *ΔNp63* expression led us to examine if any mutations were present within the coding sequence of the *TP63* gene in BEEC patients. We therefore sequenced all 15 exons of *TP63* gene [11] in 15 BEEC patients but found no mutations (data not shown). This finding confirms that of a recent study where no exon mutations were found in a study of 22 BEEC patients [14].

To explain reduced *ΔNp63* expression in the absence of any exon mutation, the *ΔNp63* promoter (2700 nucleotides upstream of exon 3) was sequenced in BEEC patients and normal controls. DNA was extracted from buccal swab samples from 163 BEEC patients and 285 ethnicity-matched controls from India, Bangladesh, China, Australia, Spain, Canada and USA. We found 7 single nucleotide polymorphisms (SNPs; 2 of which were novel ss#541026548 and ss#541027120) and 4 insertion/deletion (indel; 1 novel ss#541028600) polymorphisms in the *ΔNp63* promoter in both BEEC and control sequences (Table 2). There were no significant deviations from Hardy-Weinberg equilibrium in the controls' genotype distributions when tested by a goodness of fit χ-square test [15]. In a number of cases we were unable to obtain complete sequences from patient or control samples.

**Table 2:** Single nucleotide polymorphisms and insertion/deletion polymorphisms in *ΔNp63* promoter found in BEEC patients and normal controls.

Ch. 3 position	NCBI rs or ss# number	Promoter position	Genotype	Numbers		Frequency (%)	
				BEEC	Controls	BEEC	Controls
190987627	ss#541026548*	−2657	A/A	59	147	86.8	68.7
	Novel SNP		A/T	8	61	11.8	28.5
			T/T	1	6	1.5	2.8
190987634	rs2138247	−2651	C/C	53	137	65.4	66.5
			C/T	28	65	34.6	31.6
			T/T	0	4	0.0	1.9
190987853	ss#541027120	−2431	G/G	46	77	64.8	65.3
	Novel SNP		G/A	20	37	28.2	31.4
			A/A	5	6	7.0	3.4
190987991	rs6148242	−2293 to −2282	TCCAGAATCTTT/TCCAGAATCTTT	70	108	56.0	71.5
	12 bp del.		TCCAGAATCTTT/-	48	42	38.4	27.8
			-/-	7	1	5.6	0.7
190988024	rs1554130	−2260	C/C	23	25	74.2	78.1
			C/T	7	7	22.6	21.9
			T/T	1	0	3.2	0.0
190988340	rs55803942	−1944	C/C	33	81	64.7	47.6
			C/T	16	75	31.4	44.1
			T/T	2	14	3.9	8.2
190988997	rs5855273	−1287	-/-	51	78	47.2	57.8
	1 bp ins.		T/-	34	53	31.5	39.3
			T/T	23	4	21.3	3.0
190989073	rs1464118	−1209	T/T	29	77	54.7	44.3
			C/T	18	70	34.0	40.2
			C/C	6	27	11.3	15.5
190989223	rs1464117**	−1059	C/C	29	67	58	41.4
			A/C	13	65	26	40.1
			A/A	8	30	16	18.5
190990212	rs34201045	−71	-/-	83	95	84.7	88.8
	2 bp ins.		-/AG	15	11	15.3	10.3
			AG/AG	0	1	0.0	0.9
190990212	ss#541028600	−71	-/-	36	49	36.7	43.8
	Novel 4 bp ins.		AGAG/-	42	53	42.9	47.3
			AGAG/AGAG	20	10	20.4	8.9

Indel polymorphism/SNP rs numbers from reference sequences of *p63* GenBank (Locus: NC_000003). Sumitter SNP (ss) accession numbers from NCBI. Data were sourced from 108 Caucasian patients, 126 Caucasian controls, 55 Non-Caucasian patients and 159 Non-Caucasian controls.

* Heterozygous Novel SNP ss#541026548 (Ch. 3 position 190987627) was associated with a decreased risk of BEEC (p = 0.0071, OR = 2.357, CI 95% = 1.256–4.426).

** Heterozygous SNP rs1464117 (Ch. 3 position 190989223) was associated with a decreased risk of BEEC (p = 0.0379, OR = 2.164, CI 95% = 1.035–4.527). Data were analyzed with contingency tables, χ-square, odds ratio and 95% confidence intervals.

**Figure 2:**
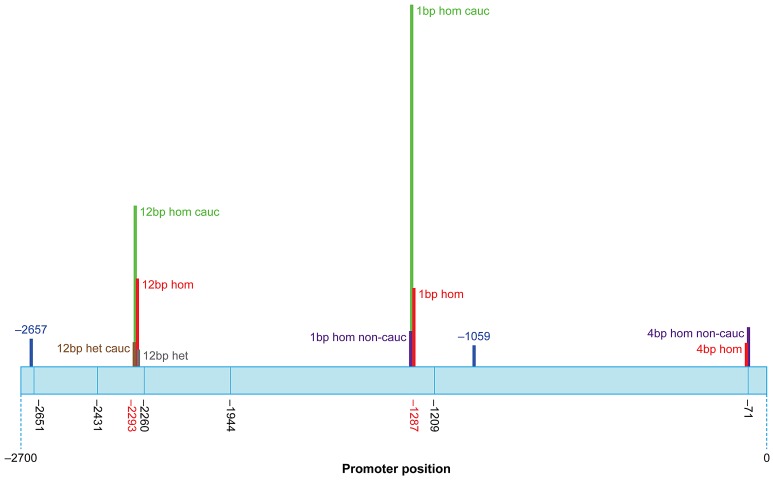
Schematic representation of the human *ΔNp63* promoter showing SNP and indel locations. The height of the bars represents the Odds Ratio (OR). Promoter positions of SNPs and indel that are not associated with any significant change in risk of BEEC are shown in black. OR represented: 12 bp heterozygous (het) all patients (grey; OR = 1.763); 12 bp homozygous (hom) all patients (red; OR = 10.80); 1 bp hom all patients (red; OR = 8.794); 4 bp hom all patients (red; OR = 2.722); 12 bp het Caucasian patients (brown; OR = 2.357); 12 bp hom Caucasian patients (green; OR = 18.33); 1 bp hom Caucasian patients (green; OR = 44.10); 1 bp hom Non-Caucasian patients (purple; OR = 4.513); 4 bp hom Non-Caucasians patients (purple; OR = 4.583). Two SNPs associated with decreased risk of BEEC are shown in blue: SNP −1059 (OR = 2.164); SNP −2657 (OR = 3.060).

Significantly, three indel polymorphisms (rs6148242, rs5855273, ss#541028600) were associated with increased risk of BEEC (Table 3). The indel polymorphisms showed no linkage disequilibrium (12 base pair (bp) deletion (del) vs. 4 bp insertion (ins), r^2^ = 0.0754; 1 bp vs 4 bp, r^2^ = 0.145; 12 bp vs 1 bp, r^2^ = 0.0273) [16]. We stratified the cohort into Caucasian and non-Caucasian groups based on the increased incidence in Caucasian populations [4]. The non-Caucasian group contained mainly Indian and Chinese patients and there were no significant differences in the three indel polymorphisms between the Indian and Chinese patients. A significant ethnicity bias of *ΔNp63* promoter base pair (bp) indel polymorphisms was observed in patients compared to their ethnically matched controls. The 12 bp deletion (rs6148242) was only associated with increased risk of BEEC among Caucasians but not in non-Caucasian patients. Conversely, the novel homozygous 4 bp insertion (ss#541028600) was found to be only associated increased risk of BEEC amongst non-Caucasians. The homozygous 1 bp insertion polymorphism (rs5855273) was significant across both cohorts (Table 4; Table 5). Interestingly, two heterozygous SNPs (ss#541026548, rs1464117) were associated with decreased BEEC risk (p = 0.0043 Odds Ratio (OR) = 3.060 and p = 0.0379 OR = 2.164 respectively) (Table 2; Figure 2).

**Table 3:** Comparison of genotype frequencies between BEEC patients and normal controls.

Indel polymorphism	Heterozygous			Homozygous		
	p	OR	95% CI	p	OR	95% CI
**12-bp Del. (rs6148242)**	0.0291	1.763	1.057–2.942	0.0068	10.80	1.300–89.73
**1-bp Ins. (rs5855273)**	0.9465	-	-	<0.0001	8.794	2.872–26.93
**4-bp Ins. (ss#541028600)**	0.8018	-	-	0.0220	2.722	1.137–6.516

Data were sourced from 108 Caucasian patients, 126 Caucasian controls, 55 Non-Caucasian patients and 159 Non-Caucasian controls. Actual numbers for each genotype are shown in Table 2. P: p-value. OR: odds ratio CI: 95% confidence intervals. Del: deletion. Ins: insertion. bp: base pair. Data were analyzed with contingency tables, χ-square, odds ratio and 95% confidence intervals.

**Table 4:** Comparison of each of the BEEC patient groups against the ethnically-matched controls.

	Caucasians vs. controls	Non-Caucasians vs. controls
**Heterozygous 12-bp deletion** **(rs6148242)**	p = 0.0071, OR = 2.357, CI: 1.256–4.426	p = 0.2325
**Homozygous 12-bp deletion** **(rs6148242)**	p = 0.0052, OR = 18.33, CI: 0.9869–340.6	p = 0.1754
**Homozygous 1-bp insertion** **(rs5855273)**	p<0.0001, OR = 44.10, CI: 2.549–762.8	p = 0.0368, OR = 4.513, CI: 1.036–19.66
**Homozygous 4-bp insertion** **(ss#541028600)**	p = 0.0892	p = 0.0259, OR = 4.583, CI: 1.160–18.10

Frequencies for each genotype are shown in Table 5. P: p-value. OR: odds ratio CI: 95% confidence intervals. Del: deletion. Ins: insertion. bp: base pair. Data were analyzed with contingency tables, χ-square, odds ratio and 95% confidence intervals.

**Table 5:** Frequency (%) of indel genotypes in Caucasian and non-Caucasian groups.

Frequency (%) of indel genotypes in Caucasian and non-Caucasian groups					
		Caucasians		Non-Caucasians	
Indel polymorphism	Genotype	BEEC	Controls	BEEC	Controls
12-bp Del. (rs6148242)	TCCAGAATCTTT/TCCAGAATCTTT	48.0	71.3	78.0	75.0
	TCCAGAATCTTT/-	45.4	28.7	17.1	25.0
	-/-	6.5	0.0	4.9	0.0
1-bp Ins. (rs5855273)	-/-	52.2	63.9	33.3	40.0
	T/-	27.5	36.0	38.4	52.5
	T/T	20.3	0.0	28.2	7.5
4-bp Ins. (ss#541025600)	-/-	20.7	50.0	15.4	35.5
	AGAG/-	50.9	43.1	50.0	45.1
	AGAG/AGAG	9.4	6.9	38.4	20.0

Data were sourced from 77 Caucasian patients, 94 Caucasian controls, 41 Non-Caucasian patients and 44 Non-Caucasian controls. Del: deletion. Ins: insertion. bp: base pair.

**Figure 3:**
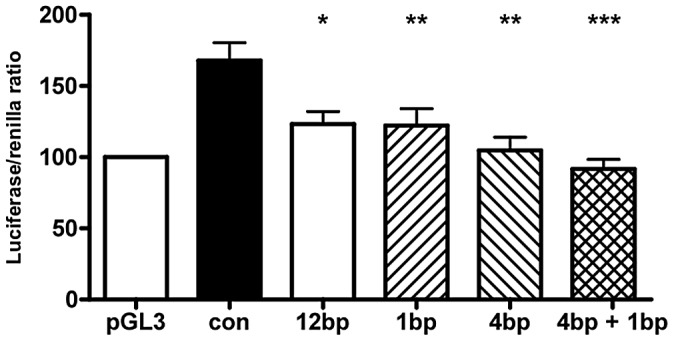
Indel polymorphisms decrease transcriptional efficiency. Luciferase assay of *ΔNp63* promoters with various indel polymorphisms transfected into human embryonic kidney (HEK-293) cells. Normal Control sequence (con), 12 base pair deletion (12 bp; rs6148242), 1 base pair insertion (1 bp, rs5855273), 4 base pair insertion (4 bp, ss#541028600). Data shown is the average of 8 experiments, each examined in triplicate. Data +/− SEM, * : p<0.05, ** p<0.01, *** : p<0.001, compared to normal control, One-way ANOVA, Bonferroni's Multiple Comparison test.

To assess the effect of different indel polymorphisms on transcriptional efficiency, we performed luciferase assays to test the *ΔNp63* promoters. We sub-cloned four variations of indel polymorphisms (Table 6) into the pGL3 luciferase vector and transfected human embryonic kidney (HEK-293) cells. We found a consistent and statistically significant reduction of the transcriptional efficiencies of the promoters containing indel polymorphisms compared with the control sequence lacking indel polymorphisms (Figure 3; Table 7).

**Table 6:** Sequences of BEEC patients and controls sub-cloned into the pGL3 luciferase vector.

	Genotype of each luciferase reporter clone				
Promoter Position	Control	12 bp (Cauc)	1 bp (Non-Cauc)	4 bp (Non-Cauc)	4 bp+1 bp (Non-Cauc)
−2657	A	A	A	A	T
−2651	C	C	C	C	C
−2293 to −2282	TCCAGAATCTT	-	TCCAGAATCTT	TCCAGAATCTT	TCCAGAATCTT
−1944	C	C	T	C	T
−1287	-	-	T	-	T
−1209	T	T	C	C	C
−1059	C	C	A	A	A
−71	-	-	-	AGAG	AGAG

Cauc: Caucasian patient sequence. Non-Cauc: Non Caucasian patient sequence. Bp: base pair.

**Table 7:** Statistical significance of reduction of transcriptional efficiencies of indel polymorphic *ΔNp63* promoters compared to normal controls.

	Human embryonic kidney cell line: HEK-293		
	P	Mean diff.	95% CI
control vs. 12 bp del.	P<0.05	44.76	6.023 to 83.50
control vs. 1 bp ins.	P<0.01	45.76	7.019 to 84.49
control vs. 4 bp ins.	P<0.001	63.21	24.47 to 101.9
control vs. 4 bp+1 bp ins.	P<0.001	76.18	37.44 to 114.9

P: p-value. CI: 95% confidence intervals. Bp: base pair. Ins: insertion, Del: deletion. Data compared to normal control, One-way ANOVA, Bonferroni's Multiple Comparison test.

A number of studies suggest a genetic component in the etiology of BEEC. Precise regulation of *ΔNp63* expression is required for development and differentiation of the ventral bladder urothelium during human development. In addition, *p63^−/−^* mice also have phenotypes identical to the associated anomalies of BEEC patients (Table 8). The only genetic model of the BEEC provides valuable insight into the possible mechanism of BEEC in humans. The mouse model showed that the anti-apoptotic isotype of p63, *ΔNp63*, is predominantly expressed in fetal murine bladder epithelium. Loss of *ΔNp63* expression in *p63^−/−^* mice increased apoptosis in the ventral bladder epithelium causing reduced mesenchymal induction (indicated by reduced expression of the mesenchymal markers Msx-1 and Fgf8) [9]. The reduction in mesenchymal cell proliferation and failure of smooth muscle formation in turn resulted in ventral midline defects, i.e. BEEC [9]. Brought together this evidence strongly suggests that *TP63* is a candidate gene for human BEEC and dysregulation of p63 expression could be a contributing factor in BEEC pathogenesis.

**Table 8:** The associated anomalies of BEEC patients compared with the *p63^−/−^* mouse phenotype.

Associated anomalies of BEEC patients	*p63^−/−^* knock-out mice phenotype
Exomphalos (88–100%)	Exomophalos [9]
Ano-rectal malformations	Ano-rectal malformations [32]
Spina bifida, sacral hypoplasia, myelomeningocele	Stunted tail [12]
Cleft palate, median cleft face syndrome [27]	Cleft palate [33]
Epidermolysis bullosa	Non-stratified skin epidermis [33]
Absence of feet, tibial deformity [27]	Absent or stunted limb buds [12]

Our murine model shows that the urogenital tubercle, the embryological origin of the foreskin, is one of few anatomical sites where *ΔNp63* is expressed [9]. Foreskin in our human study is one of p63 expressing tissues and is logistically more accessible. Our study has shown that *ΔNp63* is the dominant isotype in human foreskin, that *ΔNp63* expression was reduced in the urothelium of BEEC patients, and expression of the pro-apoptotic *TAp63* was increased. No exon mutations have been discovered in *TP63* suggesting that dysregulation may be caused by other regions of the gene such as the promoter regions. Sequence variants in promoters have been shown to be risk factors in numerous diseases including pneumoconiosis [17], auto-immune diseases [18], asthma [19], and β-thalassaemia [20]. A six-nucleotide promoter polymorphism has been described as a risk factor in multiple cancers such as lung, esophagus, stomach, colorectum, breast and cervix in Chinese populations [21]. However, studies on breast, prostate, and colorectal cancer in European and USA populations have not shown the same association with cancer risk [22]–[24]. Sequencing of the *ΔNp63* promoter region revealed three indel polymorphisms in the 163 BEEC patients associated with a statistically significant increase in BEEC risk. The prevalence and the role of indel polymorphisms differed between Caucasian and non-Caucasian ethnic populations. While further studies are required to explain this, we speculate ethnicity-specific polymorphisms of up-stream or down-stream genes may further modify the final effect of the *ΔNp63* promoter polymorphisms. One possible explanation may be that the polymorphisms may interfere with transcription binding sites which may differ in Caucasian and non-Caucasian populations. An example is the deletion of six-nucleotides in the CASP8 promoter destroys the Sp1 transcription factor binding site [21]. We searched transcription factor binding sites in the *ΔNp63* promoter using MATCH software. Computational analysis predicted the 12 bp indel might affect binding of Hand1, GATA-1,2,3,6, Gfi1, LEF1, TCF1 and SOX10, whereas the 4 bp indel may affect binding of SREBP, EGR and CBF transcription factors. Further studies would be needed to verify these suggested interactions.

In this study, indel polymorphisms in *ΔNp63* promoter are associated with increased risk of BEEC, most likely due to decreased transcriptional efficiency and therefore decreased expression of anti-apoptotic *ΔNp63* isoforms during bladder development. The consequences of decreased expression of p63 isotypes could be complex and result in stimulation or negative regulation of a number of genes. One such gene, *PERP*, is a p63 regulated gene central to epithelial integrity and homeostasis [25]. In skin deficient in PERP, desmosomal deficits are observed in addition to epithelial blistering [25]. Genome-wide expression profiling has revealed a great number of desmosomal-linked genes in addition to *PERP, SYNOP2*, and the Wnt pathway as potentially contributing to BEEC etiology [26]. P63 mutation has been implicated in human disease such as Ectodactyly, Ectodermal dysplasia and Cleft palate/lip syndrome (EEC) [27]. Maas et al., reported that, out of 14 members of a family with EEC syndrome, 10 suffered from micturition problem [28]. After reviewing 24 previous reports of urogenital anomalies in EEC patients, Maas concluded that structural anomalies of urogenital system may be part of EEC syndrome. The report included a histological figure of “atrophic” urothelium, which showed area of thin urothelium, reminiscent of the non-stratified bladder epithelium of *p63^−/−^* BEEC knockout model [28]. The association is further supported by a report from Chuangsuiwanich et al., which showed a case of EEC foetus with markedly hypoplastic bladder, lined at it lower part with thin urothelium [29]. Sub-clinical variations of BEEC may be more prevalent in EEC patients and/or other conditions than previously thought.

We conclude that insertion/deletion polymorphisms of *ΔNp63* promoter are associated with increased risk of Bladder Exstrophy Epispadias Complex. We believe our results provide a base for further study of p63 related genes in this debilitating condition

## Materials and Methods

### DNA Samples

The Research Ethics Committee of Royal Children's Hospital, Melbourne, Australia reviewed and approved the study. Informed consents were obtained from the patients, parents/guardians and the normal controls. Buccal swabs were used to collect DNA extracted with the BuccalAmp DNA Extraction Kit (Epicentre Biotechnologies, Madison, WI, USA). All samples were codified and subject identities kept confidential. Collaborators from overseas centres (Canada, USA, Spain, India, Bangladesh, China, and Malaysia) had the study approved by their institutional ethic committees prior to sample collection.

### PCR and Sequencing

Exon PCR amplification was performed using published protocols [27] and in-house primers designed using Primer 3 and Premier Netprimer (Premier BioSoft International). Four pairs of primers were designed to complete the PCR and sequencing of the 2785 bp promoter in four parts. The extent of the *ΔNp63* promoter by aligning and comparing the 5′ upstream regions of exon 3 of *P63* gene sequences from humans, mouse and pig. The most conserved region 2785 bp (−2696 to +89) was selected as the putative region of *ΔNp63*. Sequencing was carried out in both directions however in a number of cases sequences from patients or controls were unreadable using a number of different combinations of primers. The PCR products were purified and then used as templates for direct DNA sequencing by the automated ABI Prism 3100 Genetic Analyzer and the Big Dye Terminator kit (Applied Biosystems, Mulgrave, VIC, Australia). Sequences were compared with reference sequences of *p63* GenBank (Locus: NC_000003) using the BioEdit software (Ibis Biosciences). Data were analyzed with contingency tables, χ-square, odds ratio and 95% confidence intervals (GraphPad Prism, GraphPad Software, Inc.).

### Quantitative Real-Time PCR

As previously described [9], RNA was extracted from foreskin and bladder urothelial tissue samples from BEEC patients. Circumcision foreskin and normal bladder urothelium from cystectomy (cancer) specimens were used as controls. Tissue samples were snap frozen in liquid nitrogen, ground to powder, mixed with 10 µl of β-mercaptoethanol (Sigma-Aldrich Pty. Ltd, Castle Hill, NSW, Australia) in buffer and centrifuged. Samples were purified with QIAshredder (Qiagen Pty. Ltd., Doncaster, VIC, Australia). RNA was then extracted with RNeasy (Qiagen Pty. Ltd., Doncaster, VIC, Australia) or Trizol (Invitrogen, Mulgrave, VIC, Australia). cDNA synthesis was accomplished using Oligo dT (Invitrogen, Mulgrave, VIC, Australia), dNTPs (Invitrogen, Mulgrave, VIC, Australia), Superscript II First-Strand Synthesis Kit (Invitrogen, Mulgrave, VIC, Australia), and RNAse inhibitor (Invitrogen, Mulgrave, VIC, Australia). Real-time qPCR was carried out using SYBR Green (Applied Biosystems, Mulgrave, VIC, Australia) on a MJ Research Bio-Rad Chromo 4 cycler (Gladesville, NSW, Australia), according to published *TAp63* and *ΔNp63* primer sequences and published PCR conditions [30]. Relative expression will be analyzed by the Pfaffl methodology with β-actin or GAPDH as the endogenous control [31].

### Luciferase Assay

*ΔNp63* promoter sequences with indel polymorphisms were cloned into pGL3 luciferase reporter vector (KpnI/XhoI sites Promega, Madison, WI, USA). Human embryonic kidney (HEK-293) (Sigma-Aldrich Pty. Ltd, Castle Hill, NSW, Australia) was used for luciferase assays. Cells were cultured with DMEM (Invitrogen, Mulgrave, VIC, Australia), 10% fetal bovine serum at 37°C with 5% CO_2_. Cells were plated on a 96-well plate (BD Biosciences, North Ryde, NSW, Australia). The following day cells were co-transfected with 200 ng of pGL3-promoter constructs containing *ΔNp63* promoters and TK *Renilla* plasmid DNA with 5 µl Lipofectamine Transfection Reagent (Invitrogen, Mulgrave, VIC, Australia). Cells were harvested 48 hours later and cell lysates assayed for luciferase activity with the Dual Luciferase Reporter Assay system (Promega, Madison, WI, USA). Luminescent signals from the firefly and *Renilla* luciferase reactions were measured in a FLUOstar Optima luminometer (BMG Labtech Pty. Ltd., Mornington, VIC, Australia). Luciferase assay reagent was added, firefly luciferase luminescence measured, followed by Stop & Glo Reagent (Promega, Madison, WI, USA). The signal intensity from *Renilla* luciferase was used to normalize the signal from the firefly luciferase. One-way ANOVA with post-hoc Bonferroni's multiple comparisons test was applied to compare the relative expressions (GraphPad Prism, GraphPad Software, Inc.).

### Accession Numbers

The following submitter National Center for Biotechnology Information (NCBI) SNP (ss) accession numbers were assigned to the SNPs and indel observed in this study: promoter position −2657, ss#541026548; −2651, ss#541027004; −2431, ss#541027120; −2293-2282, ss#5411027737; −2260, ss#541027867; −1944, ss#541027977; −1287, ss#541028071; −1209, ss#541028198; −1059, ss#541028317; −71, ss#541028452 (2 bp); and −71, ss#5410228600 (4 bp).
